# Species interactions mediate thermal evolution

**DOI:** 10.1111/eva.12805

**Published:** 2019-05-09

**Authors:** M. Tseng, Joey R. Bernhardt, Alexander E. Chila

**Affiliations:** ^1^ Departments of Botany and Zoology, Biodiversity Research Centre University of British Columbia Vancouver British Columbia Canada; ^2^ Eawag Swiss Federal Institute of Aquatic Science and Technology Dübendorf Switzerland; ^3^ Present address: Department of Biology University of Victoria Victoria British Columbia Canada

**Keywords:** adaptation, algae, FlowCam, rapid evolution, species interactions, temperature, thermal evolution, thermal reaction norm, zooplankton

## Abstract

Understanding whether populations and communities can evolve fast enough to keep up with ongoing climate change is one of the most pressing issues in biology today. A growing number of studies have documented rapid evolutionary responses to warming, suggesting that populations may be able to persist despite temperature increases. The challenge now is to better understand how species interactions, which are ubiquitous in nature, mediate these population responses to warming. Here, we use laboratory natural selection experiments in a freshwater community to test hypotheses related to how thermal evolution of *Daphnia pulex* to two selection temperatures (12 and 18°C) is mediated by rapid thermal evolution of its algal resource (*Scenedesmus obliquus*) or by the presence of the zooplankton predator *Chaoborus americanus*. We found that cold‐evolved algae (a high‐quality resource) facilitated the evolution of increased thermal plasticity in *Daphnia* populations selected at 12°C, for both body size and per capita growth rates (*r*). Conversely, warm‐evolved algae facilitated the evolution of increased *r* thermal plasticity for *Daphnia* selected at 18°C. Lastly, we found that the effect of selection temperature on evolved *Daphnia* body size was more pronounced when *Daphnia* were also reared with predators. These data demonstrate that trait evolution of a focal population to the thermal environment can be affected by both bottom‐up and top‐down species interactions and that rapid temperature evolution of a resource can have cascading effects on consumer thermal evolution. Our study highlights the importance of incorporating species interactions when estimating ecological and evolutionary responses of populations and communities to ongoing temperature warming.

## 1 INTRODUCTION

Understanding how populations and communities are responding to ongoing rapid climate warming is one of the most pressing issues facing biologists today. Fortunately, in both ecology and evolution there has been a long history of investigating the effects of temperature on phenotypes, populations and communities (Angilletta, Steury, & Sears, 2004; Bennett, Dao, & Lenski, 1990; Bernhardt, Sunday, & O'Connor, 2018; Bochdanovits, Jong, & Jong, 2003; Kingsolver & Huey, 2008; Loboda, Savage, Buddle, Schmidt, & Høye, 2018; Mousing, Ellegaard, & Richardson, 2014; Newman, 2004; O'Connor et al., 2007; Patrick, 1971; Régnière & Nealis, 2002; Tseng et al., 2018; Tseng & O'Connor, 2015). These and many other ecological and evolutionary studies of temperature responses have collectively demonstrated that temperature affects all levels of organization in the natural world and that evolutionary responses of focal populations to the thermal environment can occur at ecological time scales.

Although evolutionary responses to temperature have been documented in a range of taxa including fruit flies, stickleback, mustard plants, zooplankton, lizards, fish parasites and phytoplankton (Anderson, Inouye, McKinney, Colautti, & Mitchell‐Olds, 2012; Barrett et al., 2010; Bochdanovits et al., 2003; Campbell‐Staton, Edwards, & Losos, 2016; Hendry, 2017; James & Partridge, 1995; Mazé‐Guilmo, Blanchet, Rey, Canto, & Loot, 2016; Padfield, Yvon‐Durocher, Buckling, Jennings, & Yvon‐Durocher, 2016; Schaum et al., 2017; Tseng & O'Connor, 2015), most estimates of thermal evolution have been made on populations in the absence of any species interactions. Consequently, although biotic interactions such as those between predators and prey, consumers and resources, competitors, and hosts and parasites are ubiquitous in nature, we have little data showing the importance of these interactions for how a focal population responds to the thermal environment.

Bottom‐up species interactions, such as those between resources and their consumers, can affect thermal responses of consumers if the resources themselves are rapidly evolving to temperature. For example, resources that occupy the lower rungs of aquatic and terrestrial food chains (e.g., phytoplankton, microbes and insects) are also the organisms mostly likely to evolve rapidly, and these trait changes may have important consequences for thermal evolution of higher trophic levels. Of particular global importance are the trophic consequences of phytoplankton thermal evolution. These microscopic organisms fix over 50% of the planet's carbon dioxide, they produce over 50% of the Earth's oxygen, and they are the primary source of nutrients such as proteins and essential fatty acids, which cascade up the aquatic food web and that are ultimately consumed by humans (Finkel et al., 2010).

Conversely, top‐down species interactions, such as those between predators and prey (for consistency, here we will refer to the prey as consumers), can also affect thermal evolution if predators depress the consumer population below which evolutionary responses are possible, or if predators preferentially eat consumers that happen to be particularly bad, or good, at withstanding the change in temperature (Osmond et al., 2017). Given the speed with which climate is warming around the world, there is a pressing need to better understand how these common species interactions affect thermal evolution. Here, we use a cosmopolitan freshwater food web to investigate the role of bottom‐up (phytoplankton thermal evolution) and top‐down (presence of a voracious predator) species interactions on rapid thermal evolution in the zooplankton *Daphnia pulex (*Cladocera: Daphniidae, Leydig 1860).

Our tri‐trophic study system consists of the phytoplankton *Scenedesmus obliquus* (Chlorococcales: Scenedesmaceae, Kützing, 1833, also known as *Tetradesmus obliquus* and *Acutodesmus obliquus*), *Daphnia pulex,* and larvae of the predatory midge *Chaoborus americanus* (Diptera: Chaoboridae; De Haan 1849). We use controlled laboratory experiments to assess the effects of a) *S. obliquus* thermal evolution, and b) predator presence, on *Daphnia* thermal reaction norm (TRN) evolution in response to two temperature selection treatments (12 and 18°C).

### 1.1 Predictions

Given their rapid doubling time (1–2 days) and large population size during the experiment (~500,000), we expected *S. obliquus* to show rapid thermal evolution.

This temperature evolution manifests as shifts in cell size, whereby cell size decreases as temperature increases (Chen, Li, Dai, Sun, & Chen, 2011; Margalef, 1954). *Scenedesmus obliquus* also produce fewer essential fatty acids at warmer temperatures, and warm‐reared *S. obliquus* sustain smaller populations of *Daphnia* compared to cold‐reared algae (Breuer, Lamers, Martens, Draaisma, & Wijffels, 2013; Sikora, Dawidowicz, & Elert, 2014; Vigeolas et al., 2012). We thus expect warm‐evolved *S. obliquus* to be a lower‐quality food resource for *Daphnia,* and we propose two hypotheses for how phytoplankton thermal evolution may affect ecological and evolutionary responses of *Daphnia* to temperature‐mediated selection (Box , Hypotheses 1–2). Cold‐evolved (higher quality) algae could facilitate *Daphnia* thermal reaction norm (TRN) evolution if this food type sustains higher populations of *Daphnia,* thus maintaining the standing genetic variation on which thermal selection can act (Frankham, 1996). Concomitantly, if a lower‐quality food source (warm‐evolved algae) imposes selection for *Daphnia* genotypes that can better withstand this food environment, the stressful environment may erode the available genetic variation (Charmantier & Garant, 2005; Kause et al., 2001), resulting in a muted response of *Daphnia* to temperature selection (Box , Hypothesis 1 (a) versus (b)). Conversely, because zooplankton and phytoplankton tend to be exposed to similar abiotic environments in nature, it is also possible that we will see local adaptation, whereby cold‐selected *Daphnia* evolve higher performance when they are fed with phytoplankton from a similar environment, and vice versa for warm‐reared *Daphnia* (Box , Hypothesis 2, (c) versus (d)). Here, we have assayed thermal reaction norm evolution so that we can quantify the relative roles of phenotypic plasticity versus evolutionary change in *Daphnia* thermal responses.

Box 1 **Potential bottom‐up effects of resource (algae) evolution on thermal reaction norm (TRN) evolution of consumers (*Daphnia*)**

1

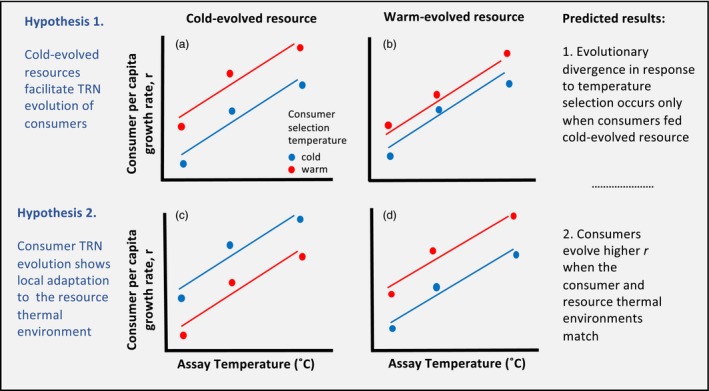

We also examined whether the presence of predators mediated evolutionary responses of *Daphnia* to the thermal environment (Box ). Here, we propose two possible outcomes (Hypothesis 3.1, 3.2). In the presence of predators, classical life‐history theory predicts that consumers should evolve higher overall per capita growth rate (Abrams & Rowe, 1996) (Box , Hypothesis 3.1 (a) versus (b)). Secondly, *Chaoborus* larvae are known to be voracious predators of *Daphnia,* and thus, predator presence may reduce *Daphnia* population size (and thus genetic variation) to levels below which thermal evolution is feasible (Hypothesis 3.2 (a) versus (c)).

Box 2 **Potential top‐down effects of predators (*Chaoborus* larvae) on thermal reaction norm (TRN) evolution of consumers (*Daphnia*)**

1

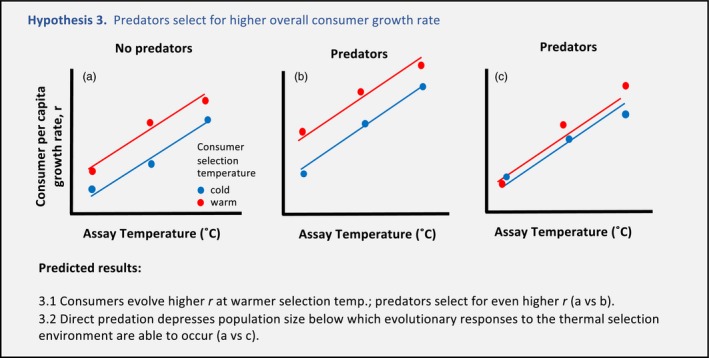

## 2 MATERIALS AND METHODS

Approximately 5,000 *Daphnia pulex* and 100 third‐instar *Chaoborus americanus* were collected from the University of British Columbia Research Ponds in early November 2016 (average temperature 8°C). *Daphnia* and *Chaoborus* were brought back to UBC and held at 12°C in a walk‐in environmental chamber. *Daphnia* were fed a laboratory culture of *Scenedesmus obliquus*, and *Chaoborus* were fed wild‐collected *Daphnia*. *Scenedesmus obliquus* (CPCC 5) was obtained from the Canadian Phycological Culture Centre in 2015 and maintained in axenic cultures at 20°C, under light‐ and nutrient‐ saturated conditions in Bold's Basal Medium (Bold, 1949). After 4 weeks of acclimation to the laboratory environment, 30 *Daphnia* were haphazardly allocated to each of 160 glass jars (750 ml) filled with 700 ml COMBO media (Kilham, Kreeger, Lynn, Goulden, & Herrera, 1998). One third‐instar *Chaoborus* was placed into half of the jars (*n* = 80). Half of the 160 jars were then placed in 12°C water baths (40 jars with predators, 40 without), and the other half were placed in 18°C water baths (40 jars with predators, 40 without). For the 18°C treatment, the temperature of the water bath was gradually raised from 12 to 18°C over a period of 1 week. To minimize environmental variation across water baths, each week all *Daphnia* population jars were haphazardly moved among water baths of the same temperature.

### 2.1 Algae treatments and maintenance

Sixteen replicate populations of algae were seeded using the laboratory culture of *Scenedesmus obliquus*. Populations were grown in COMBO medium in 1.9‐L glass jars. Jars were capped and aerated using filtered air (0.3‐µm in‐line HEPA‐VENT filter) from a large aquarium air pump. Eight replicate jars were maintained at 12°C (cold‐algae treatment), and eight were maintained at 18°C (warm‐algae treatment).

Three times per week, approximately 3/5 of the contents of each of the eight algal populations per temperature treatment were poured out into a single bucket. Individual algal jars were refilled with COMBO and returned to the water bath. The pooled contents from each temperature treatment were fed to the appropriate *Daphnia* populations. The pooling of algae across the eight populations per temperature treatment for *Daphnia* feeding was purposeful because we wanted *Daphnia* to be exposed to generic “cold‐reared” or “warm‐reared” algae. Algal replicate jars remained separate for quantification of experimental evolution.

At four occasions during the experiment, we quantified algal cell size, and the number of algal cells after dilution, and immediately prior to the following dilution (typically 2 days later), using a FlowCam imaging particle counter (FlowCam^®^ VS Series; Fluid Imaging Technologies), at a flow rate of 0.3 ml/min. We calculated population growth rate during each period as cell number at time *t* + 1 divided by cell number at time *t*. There was no difference in population growth rate between algae maintained at the two temperature treatments (growth rate = 0.43 cells/day; temperature × date *F*_1,124_ = 0.17, *p* = 0.68). This lack of difference in growth rate is perhaps surprising because we typically believe that algae grow faster at warmer temperatures. We have since confirmed this result by growing the same strain of *S. obliquus* across multiple temperatures without dilution and found that differences in growth rate are not detectable until after the third day of continuous growth (data available upon request). Finally, at the beginning of the experiment we also used haemocytometer counts to confirm density estimates generated by the FlowCam, and found little difference between the estimates of cell density obtained using the two methods (FlowCam: 402,366 cells/ml; haemocytometer: 454,000 cells/ml).

### 2.2 *Daphnia* maintenance

*Daphnia* were fed either cold‐ or warm‐reared algae for the duration of the experiment. The number of algal cells per feeding was quantified using FlowCam, and we converted cells per feeding to carbon per feeding using published biovolume to biomass conversion equations (Montagnes, Berges, Harrison, & Taylor, 1994). Algal carbon per feeding (16 mg C/L per day) exceeded the amount typically fed to *Daphnia* to maintain saturating levels of algae (1–2 mg C/L) (Horton, Rowan, Webster, & Peters, 1979; Koch, Martin‐Creuzburg, Grossart, & Straile, 2012; Weider, Jeyasingh, & Looper, 2008; West & Post, 2016). *Daphnia* populations were given a 100 ml COMBO change once per week (~14% media change). Overall, this was a three‐factor experiment, with two levels of predators (present/absent), two levels of *Daphnia* rearing temperature (12°C, 18°C) and two levels of algal food type (cold, warm). There were twenty replicate *Daphnia* populations per treatment combination for a total of 160 replicate populations.

### 2.3 Data collection

#### 2.3.1 *Daphnia* population size

*Daphnia* population size was censused on days 1, 15, 45, 75, 105 and 150 of the experiment. The contents of each replicate jar were poured into a sorting tray, and all *Daphnia* within each jar were manually counted by returning individual Daphnia to replicate jars using a plastic disposable pipette. Approximately 76,000 individual *Daphnia* were counted over the duration of the experiment.

#### 2.3.2 Common garden environment

On day 150 of the experiment, all predators were removed, and the temperature of all *Daphnia* and algae populations was set to 15°C (common garden environment). Day 150 represents approximately 10–15 rounds of reproduction in *Daphnia* and ~65 cell division cycles for *S. obliquus*. *Daphnia* have overlapping generations. It takes 10–15 days to go from neonate to neonate at the two temperatures used in this experiment, and mature adults continue to produce a new brood every 3–5 days until death. *Daphnia* were fed the laboratory stock of *S. obliquus* from day 150 onward.

#### 2.3.3 Assaying evolved differences in algal respiration and photosynthesis

After two rounds of algal replication in the common garden environment, we assayed cell volume and rates of photosynthesis and respiration, across a range of temperatures. We used the FlowCam to quantify average individual algal cell volume from a 0.3 ml sample from each replicate population. *Scenedesmus obliquus* exhibits morphological variation, whereby cells can either exist as unicells or colonies (coenobia, a group of daughter cells that share a parent cell wall) (Lürling & Van Donk, 1996). Our measure of cell volume does not distinguish between these two forms; we define “volume” as the total volume of material within the cell wall.

Rates of photosynthesis and respiration from subsamples of each replicate jar were assayed using a sealed glass microplate in combination with a microplate oxygen sensor. The microplate contained 24 x 200 µl chambers each equipped with an oxygen sensor spot (Loligo Systems). The microplate was connected to a 24‐channel optical fluorescence oxygen system (SDR SensorDish Reader; Presens). The reader and microplate were placed in a temperature‐controlled incubator (Panasonic MIR‐154). Algal photosynthesis and respiration were assayed at five temperatures (10, 12, 16, 20 and 24°C). Rates of photosynthesis were estimated by measuring rates of oxygen evolution in the light and consumption in the dark. 200 µl of well‐mixed *Scenedesmus* populations from each replicate population was transferred to each well on the microplate, and measurements of oxygen concentration were taken every 15 s over 3‐hr periods, first in darkness and then in light. Wells were sealed with transparent PCR Film (Thermo Scientific). Prior to oxygen flux measurements, sensor spots were calibrated with air‐saturated water (100% oxygen) and water containing 2% sodium sulphite (0% oxygen) at each experimental temperature. Algal cells were acclimated to the assay temperature for 1 hr in the dark prior to measurements. Three blank wells with COMBO medium were run at the same time as the phytoplankton at each temperature, and the average rate of oxygen flux in these wells was subtracted from the experimental wells to account for background microbial respiration. Gross photosynthesis was estimated as GP = net photosynthesis + respiration at each temperature. We assumed that net photosynthesis was directly proportional to oxygen production in the light. We mass‐normalized rates of photosynthesis using the mean population biovolume (mean cell volume × cell density) from the source populations, measured using the FlowCam (flow rate = 0.3 ml/min) on the day of the respirometry assays. Cell volume (µm^3^) was determined with area‐by‐diameter estimation (ABD) in each replicate population.

#### 2.3.4 Assaying evolved differences in *Daphnia* life‐history traits

After 3 weeks in the common garden environment, four juvenile *Daphnia* from each replicate jar were placed in individual 50‐ml Falcon brand centrifuge tubes. Once one of the four *Daphnia* matured and produced at least four neonates, the remaining three *Daphnia* were discarded. These four neonates were transferred to individual 50‐ml Falcon tubes filled with 40 ml COMBO and placed in one of four assay temperatures: 12, 18, 22 or 26°C. Because these offspring were produced parthenogenetically, each of the 160 replicate *Daphnia* populations thus contributed four genetically identical individuals to the evolution assay. We chose to use one *Daphnia* per replicate population in order to avoid pseudoreplication. To be statistically conservative, we used “jar” as the unit of replication for the experiment. We included 20 replicate jars (selection lines) per treatment, which is at the highest end of the number of replicates typically used for this type of experimental evolution study (Bochdanovits et al., 2003; Huang, Tran, & Agrawal, 2016; Padfield et al., 2016; Schaum et al., 2017; Van Doorslaer, Stoks, Jeppesen, & Meester, 2007).

*Daphnia* in the evolution assay were maintained on laboratory *S. obliquus* stock cultures. We checked *Daphnia* daily for eggs, and once eggs were visible, *Daphnia* were removed and preserved in 95% ethanol and photographed at 10× using an inverted microscope (Leica MC120‐HD). We recorded the number of days to first clutch (development time) and measured adult size and offspring number. The latter two traits were measured using the measuring tool in the Leica Application Suite software (version 4.4). *Daphnia* adult size (mm) was measured as the length of the body from the top of the head above the eyespot to the base of the tail spine. To measure egg number, we counted the number of eggs visible from a side‐view photograph of the ethanol‐preserved *Daphnia*. This measure is likely an underestimate of the actual egg number as we were not able to count any eggs that were hidden under the top visible layer of eggs. We used egg number to calculate *Daphnia* per capita growth rate, *r*, using the following equation: r = ln(viable eggs in first clutch)/time to first clutch; Trubetskova & Lampert, 2002).

### 2.4 Data analysis

#### 2.4.1 Algal evolution

We used linear mixed‐effects models (R package lmer) to evaluate whether *S. obliquus* photosynthesis or respiration rates evolved in response to rearing temperature. In these statistical models, selection temperature (12°C, 18°C) and assay temperature (10, 12, 16, 20, 24°C) were included as fixed effects, and “replicate” was included as a random effect. We assessed the statistical significance of the treatments and interactions using likelihood ratio tests, following Winter (2013). For the statistical analysis of algal cell volume, each replicate contributed one mean value to the analysis, and we used ANOVA to test whether rearing temperature explained variation in evolved cell volume.

#### 2.4.2 *Daphnia* population size and evolution

We used ANOVA to assess whether selection temperature (12°C, 18°C), predators (present/absent) and food type (cold vs. warm algae) explained significant variation in mean *Daphnia* population size.

We used linear mixed‐effects models (R package lmer) to examine whether rearing temperature, predators, food type and assay temperature (12, 18, 22, 26°C) explained variation in *Daphnia* evolved size at maturity, development time and per capita growth rate, *r*. Assay temperature was modelled as a continuous variable so that we could compare slopes of the thermal reaction norms among treatments. Because each replicate population jar contributed one *Daphnia* genotype (4 individuals) to the evolution assays, we included a “family” random effect in each of the models. We incorporated trait variation arising from drift among replicates by including random slopes and intercepts for the “family” term. This approach allows us to examine the variance in traits attributable to treatment effects after among‐replicate variance is accounted for (Dutilleul et al., 2014).

We  included all main effects and interaction terms in the full model for each dependent variable (main effects: food type, selection temperature, assay temperature, predator). We assessed the statistical significance of interaction terms using the ANOVA command in R (e.g., ANOVA model]), and we removed interactions terms if they were not statistically significant. We kept nonsignificant two‐way interactions in the model if a three‐way interaction that incorporated the two‐way interaction was statistically significant. We had no a priori reason to expect significant statistical interactions between food type and predator presence, and none of these interactions were significant. All statistical analyses were conducted in R v.3.4.1 (R Core Team, 2017), and all raw data are available on Dryad (https://doi.org/10.5061/dryad.d6f6q7p).

## 3 RESULTS

### 3.1 Algal responses to temperature

#### 3.1.1 Cell size

When assayed after two generations in the common garden environment (15°C), algal populations that had been maintained for the duration of the experiment at the warmer temperature were 23% smaller in volume compared to algae maintained at the cooler temperature (12°C: 1,065 µm^3^ ± 91 *SD*; 18°C: 821 µm^3^ ± 145; *F*_1,14_ = 16, *p* < 0.001). We observed a similar pattern of cell volume during the experiment, with algae reared at the warmer temperature exhibiting a 25% decrease in cell size (776 µm^3^ ± 93) compared to algae reared at the cooler temperature (1,041 µm^3^ ± 104; average of sampling days 25, 75, 105). Algal cell volumes recorded after the common garden period were not statistically different from the average cell volume during the experiment (*F*_1,60_ = 1.3, *p* = 0.26).

#### 3.1.2 Metabolism

**Figure 1:**
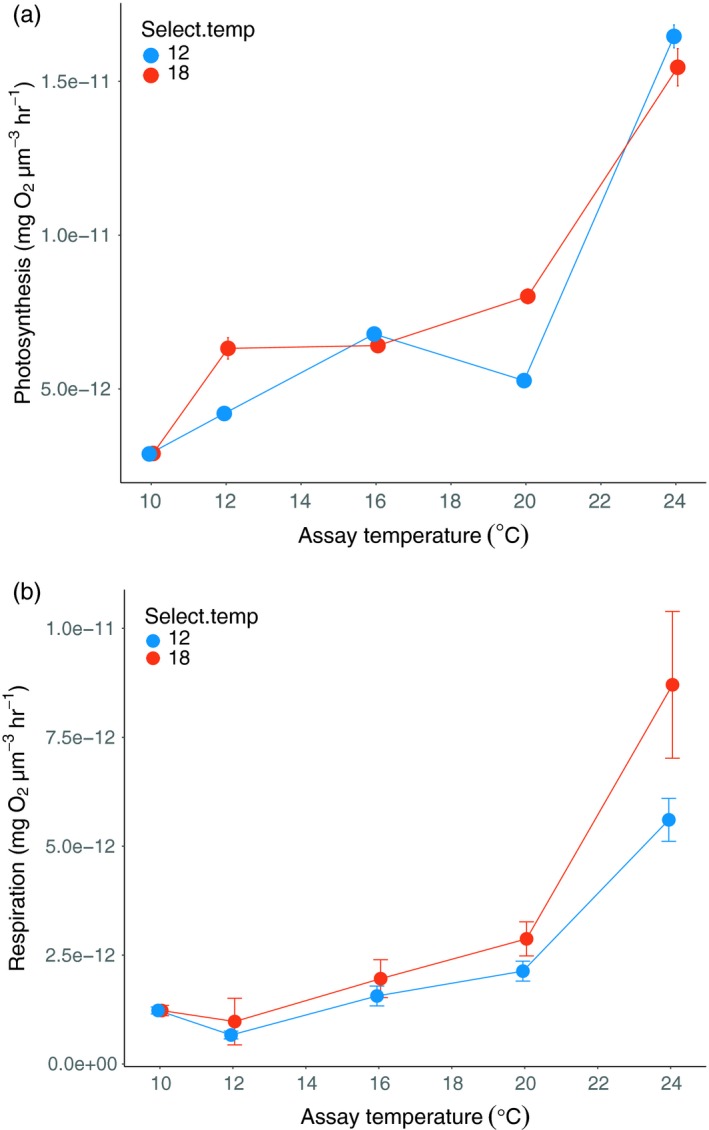
Thermal reaction norms for *Scenedesmus obliquus* (a) photosynthesis and (b) respiration. There was no effect of selection temperature on photosynthesis. Algae reared at 18°C evolved higher rates of respiration than those reared at 12°C. Both photosynthesis and respiration increased with assay temperature. See Section 3.1.2 for statistics. Blue symbols denote algae selected at the cooler temperature, and red symbols represent algae selected at the warmer temperature. Error bars represent ± 1 standard error

Rates of *S. obliquus* photosynthesis showed plastic increases with assay temperature, but there was no difference in photosynthesis between the temperature selection lines (Figure 1a.; assay temperature: *χ* ^2^(4) = 100.3, *p* < 0.0001; selection temperature: *χ* ^2^(1) = 1.1, *p* = 0.30; assay temperature × selection temperature: *χ* ^2^(4) = 5.5, *p* = 0.24).

Algal respiration also increased plastically with assay temperature (Figure 1b; *χ* ^2^(4) = 66.7, *p* < 0.0001), and *S. obliquus* populations selected at the warmer temperature evolved higher rates of respiration compared to populations selected at the colder temperature (Figure 1b; selection temperature: *χ* ^2^(1) = 4.1, *p* = 0.04). The effect of selection temperature did not depend on assay temperature (*χ* ^2^(4) = 5.98, *p* = 0.2).

### 3.2 *Daphnia* population size

**Figure 2:**
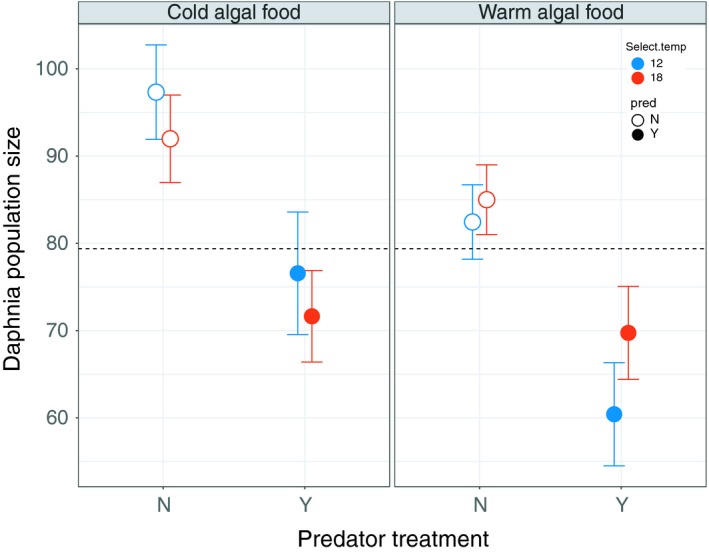
Effect of food type, predators and selection temperature on mean *Daphnia* population size. *Daphnia* fed cold‐evolved algae maintained a 12% higher overall population size compared to those fed warm‐evolved algae, and predators reduced *Daphnia* population size by 22% (see Section 42.3.3 for statistics). Error bars are ± 1 standard error

Both food type and predator treatment explained significant variation in *Daphnia* population size (Figure 2; predators: *F*_1,152_ = 33.6, *p* < 0.0001; food type: *F*_1,152_ = 8.7, *p* = 0.004). *Daphnia* populations fed warm‐reared algae exhibited a 12% lower population size (mean = 74) compared to populations fed cold‐reared algae (mean = 84; Figure 2), and the presence of predators decreased the average *Daphnia* population size by 22% (mean population size with/without predators = 89/70, respectively.

### 3.3 *Daphnia* thermal reaction norms and evolution

#### 3.3.1 Body size

**Figure 3:**
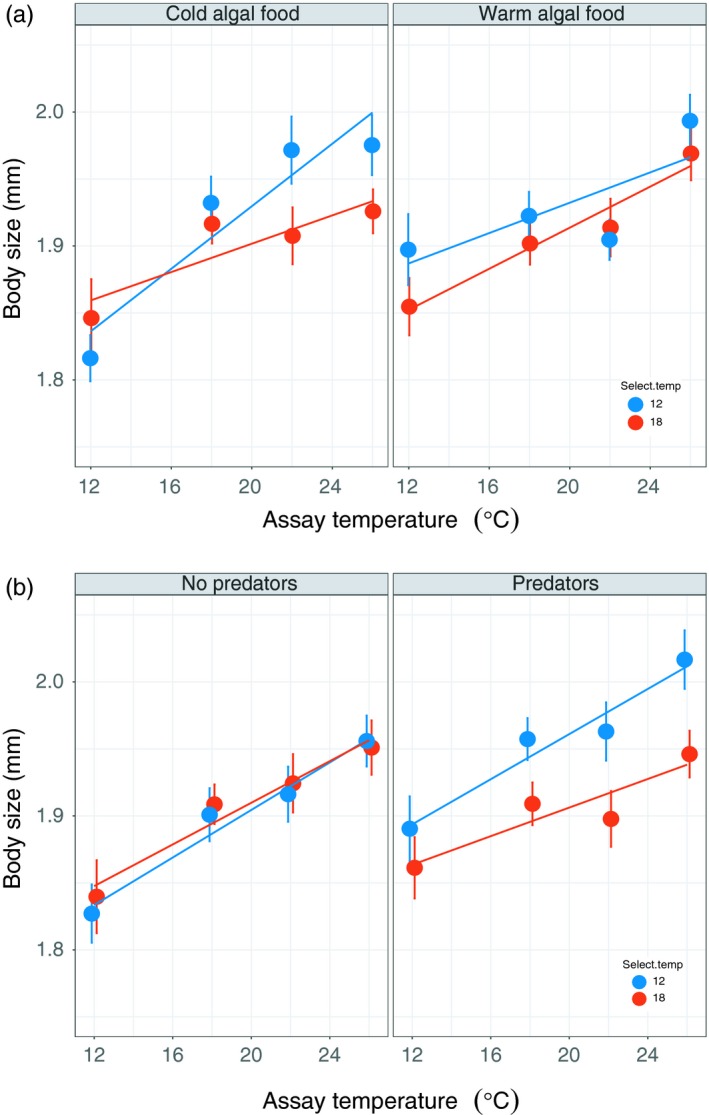
Effect of (a) algal food type and assay temperature, and (b) predators and assay temperature on *Daphnia* body size thermal reaction norm evolution. (a) *Daphnia* fed cold‐evolved algae diverged evolutionarily in response to selection temperature. However, there was no effect of selection temperature when *Daphnia* were fed warm‐evolved algae. (b) *Daphnia* reared in the presence of predators evolved larger bodies in the cold selection environment. In the absence of predators, there was no difference in body size TRN between the two temperature selection treatments. See Table 1 for statistics, error bars are ± 1 standard error

Algal food type altered *Daphnia* body size evolution in response to selection temperature (Figure 3a and Table 1). *Daphnia* fed cold‐evolved algae evolved steeper thermal reaction norms when selected at 12°C, but there was no difference in TRNs for body size in *Daphnia* fed warm‐evolved algae. There was a phenotypically plastic effect of assay temperature on *Daphnia* body size (Table 1), with *Daphnia* assayed at warmer temperatures showing slightly larger body sizes.

**Table 1:** Results of the linear mixed‐effects model examining the effect of temperature selection, predator selection and food type on *Daphnia pulex* body size, time to maturity and per capita growth rate, *r*

Factor	*F* value, *p*		
	Body size	Time to maturity	*r*
Selection temperature	0.21, 0.65	0.84, 0.36	0.15, 0.7
Food	1.14, 0.29	0, 0.95	0.67, 0.42
Predator	3.94, 0.049	3.5, 0.06	1.16, 0.28
Assay temperature	62, **<0.0001**	938, **<0.0001**	70.1, **<0.0001**
Select temperature × food	3.74, **0.05**		4.27, **0.04**
Selection temperature × predator	5.88, **0.02**		
Select temperature × assay temperature	1.3, 0.25		0.02, 0.89
Food × assay temperature	0.9, 0.34		0.55, 0.48
Select temperature × food × assay temperature	4.8, **0.03**		7.4, **0.007**

Note

Bold font denotes statistically significant terms.

The presence of predators modified how *Daphnia* body size evolved in response to the thermal environment (Figure 3b and Table 1). The effect of temperature selection was more pronounced when *Daphnia* were also reared with predators, with *Daphnia* evolving overall larger bodies in the 12°C versus 18°C selection treatments.

#### 3.3.2 Time to maturity

**Figure 4:**
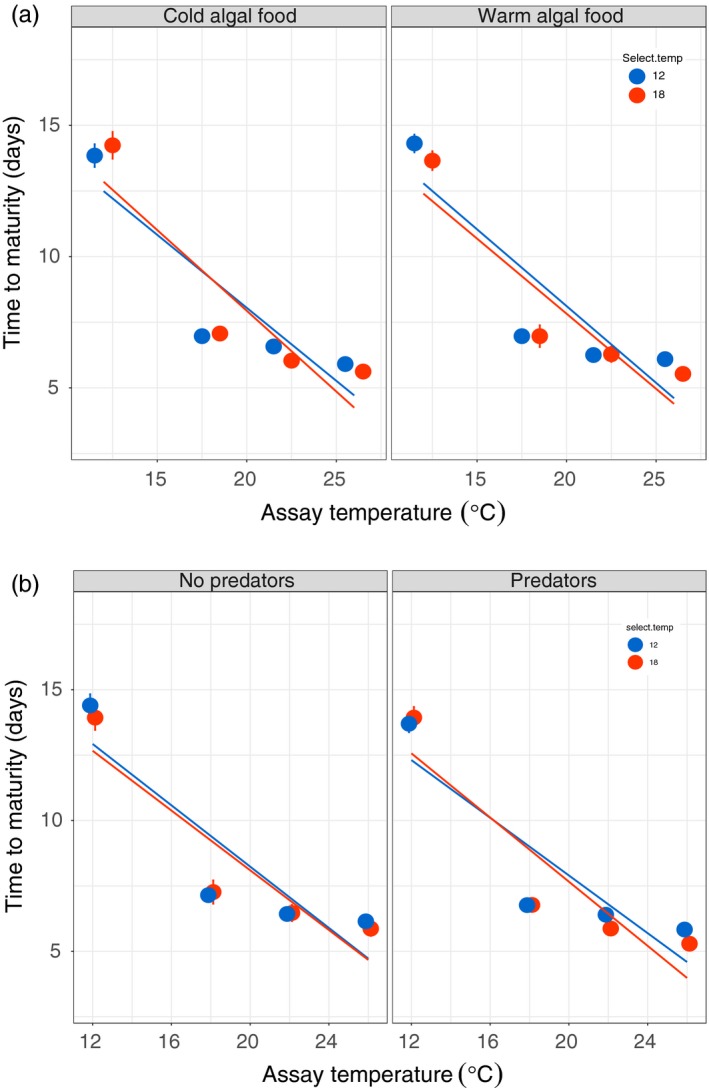
Effect of (a) algal food type and assay temperature, and (b) predators and assay temperature on *Daphnia* development time thermal reaction norm evolution. Only assay temperature significantly explained variation in development time (Table 1). Error bars are ± 1 standard error

Variation in *Daphnia* development time was primarily explained by assay temperature, with *Daphnia* assayed at warmer temperatures maturing several days earlier than those assayed at cooler temperatures (Figure 4a and Table 1). There was no effect of selection temperature or algal food type on TRN evolution. There was also no effect of predator presence on *Daphnia* development time TRNs (Figure 4b).

#### 3.3.3 Per capita* growth rate, r*

**Figure 5:**
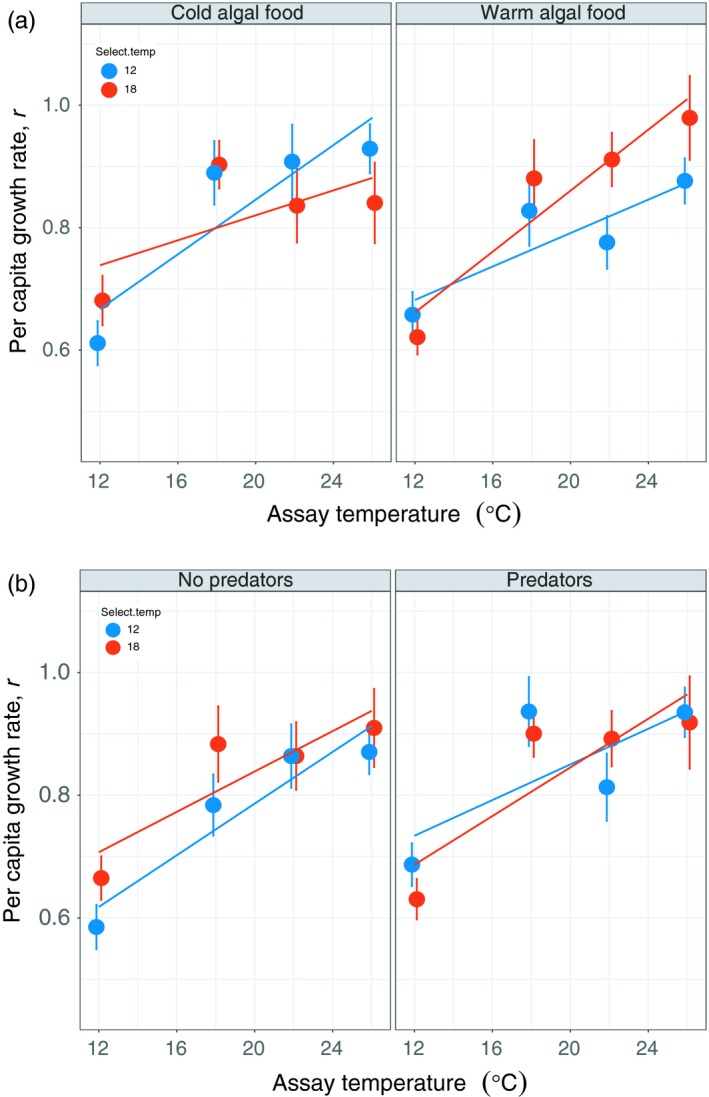
Effect of (a) algal food type and assay temperature, and (b) predators and assay temperature on *Daphnia r* thermal reaction norm evolution. At the warmer assay temperatures, *Daphnia* reared at 12°C evolved higher r when fed cold‐evolved algae, and *Daphnia* reared at 18°C evolved higher r when fed warm‐adapted algae (Table 1). (b) The presence of predators did not affect *Daphnia* r thermal reaction norm evolution. Error bars are ± 1 standard error

Algal food type altered how TRNs for *Daphnia r* evolved in response to selection temperature (Figure 5a and Table 1). When fed cold‐evolved algae, *Daphnia* evolved steeper TRNs in the 12°C selection treatment, but when fed warm‐evolved algae, *Daphnia* evolved steeper TRNs in the 18°C selection treatment (Figure 5a and Table 1). Assay temperature explained a significant fraction of variation in *Daphnia r*, with *Daphnia* exhibiting faster *r* when grown in warmer assay temperatures. There was no effect of predators on TRN evolution in *Daphnia r* (Figure 5b and Table 1).

## 4 DISCUSSION

The goal of this study was to investigate the effect of bottom‐up and top‐down species interactions on thermal evolution of a focal zooplankton population. We documented increases in phytoplankton cell volume in response to the colder rearing environment from early on the experiment and confirmed that these changes had an evolutionary basis at the end of the experiment. We demonstrated that *Daphnia* fed cold‐evolved algae maintained larger population sizes compared to populations fed warm‐evolved algae. *Daphnia* fed cold‐evolved algae evolved steeper TRNs for body size, and *Daphnia* reared in the presence of predators evolved overall larger bodies. Lastly, the evolution of TRNs for *Daphnia* per capita growth rate was driven by bottom‐up species interactions, with *Daphnia* fed cold‐evolved algae exhibiting steeper TRNs in the cold *Daphnia* selection environment, and vice versa for *Daphnia* fed warm‐evolved algae. We discuss these results in further detail below.

### 4.1 Algal evolution

Algae exhibit cell size plasticity in response to temperature (Chalifour & Juneau, 2011; Chen et al., 2011; Margalef, 1954; Rhee & Gotham, 1981), and thus, differences in cell size between the two temperature selection experiments were likely a function of both plasticity and evolution. The data collected here do now allow us to quantify exactly when in the experiment algal evolution occurred. However, using population sizes less than those used in this experiment (~100,000 cells/ml), we have since demonstrated that *S. obliquus* can evolve to the thermal environment in <2 weeks (M Tseng, E. Yangel, A. Zhou, unpublished manuscript).

Two recent studies on thermal reaction norm evolution in other species of phytoplankton also reported the evolution of elevated respiration in warmer selection environments, but in both of these studies, they also saw the evolution of increased photosynthesis (*Chlorella vulgaris*: Padfield et al., 2016, *Chlamydomonas reinhardtii*: Schaum et al., 2017). The duration of selection was 100 generations, and 10 years, for *Chlorella*, and *Chlamydomonas,* respectively, suggesting that the 70 generations used here may have been too short to observe photosynthesis evolution. However, the evolved increase in resource consumption (respiration) but not resource acquisition (photosynthesis) at warmer temperatures is consistent with the observed evolved decrease in cell size. Finally, although not directly tested here, cell size is positively correlated with lipid content in other algal species (Bono, Garcia, Sri‐Jayantha, Ahner, & Kirby, 2015). This pattern suggests a possible mechanistic link between cold temperature, cell size, increased algal fatty acids and increased nutritional quality of cold‐reared algae.

### 4.2 Effect of resource thermal evolution on *Daphnia* population size

Our results demonstrated that algal thermal evolution had significant effects on *Daphnia* population size. *Daphnia* maintained on cold‐reared algae attained a 12% higher population size during the experiment compared to *Daphnia* maintained on warm‐reared algae. These results are consistent with a previously published study showing that cold‐reared *Scenedesmus obliquus* are more nutritious and sustain higher *Daphnia* population sizes, compared to warm‐reared *S. obliqu*us (Breuer et al., 2013; Sikora et al., 2014; Vigeolas et al., 2012).

### 4.3 Effect of resource evolution on *Daphnia* thermal evolution: “bottom‐up effects”

From previous studies demonstrating a positive correlation between environmental quality and genetic variation/heritability (Bochdanovits et al., 2003; Charmantier & Garant, 2005), we predicted that *Daphnia* reared long term on cold‐evolved (higher quality) phytoplankton would show an elevated evolutionary response to temperature‐mediated selection (Box , Hypothesis 1). However, we also predicted that because zooplankton and phytoplankton often encounter the same abiotic environment in nature, a “better” environment might be one in which the consumer environment matches that of the resource (Box , Hypothesis 2). Our data were consistent with both hypotheses. (a) *Daphnia* body size evolution in response to selection temperature was more pronounced in populations that were fed the cold‐evolved/higher quality algae, consistent with Hypothesis 1 (Figure 3a); (b) conversely, *Daphnia r* evolution exhibited patterns indicative of local adaptation between the consumer and resource thermal environment, consistent with Hypothesis 2 (Figure 5a). What we did not predict at the outset of this study was that rapid algal evolution could potentially change the slope of the evolved TRN. A steeper TRN slope reflects an overall increase in thermal plasticity. It is unclear why *Daphnia* in these “matched” (e.g., cold‐evolved algae + cold‐selected *Daphnia*) environments would show elevated thermal plasticity, compared to *Daphnia* in the “mismatched” environments. However, these patterns are consistent with previous studies showing the expression of steeper TRNs when organisms were fed higher quality food (Álvarez & Nicieza, 2002; Jang, Rho, Koh, & Lee, 2015; Kingsolver, Shlichta, Ragland, & Massie, 2006).

### 4.4 Potential for maladaptation

One would predict that organisms with rapid generation times (e.g., phytoplankton or microbes) will evolve much more quickly to warming, relative to their consumers (e.g., zooplankton), which have longer generation times and smaller population sizes. As temperatures in nature continue to warm, the mismatch in rates of thermal evolution between resources and consumers could result in slower‐evolving consumers interacting with resources that have already evolved to a changed environment. This hypothetical situation is not totally dissimilar from the “warm‐evolved algae + cold‐selected *Daphnia*” treatment combination used in this study. For both body size and *r*, *Daphnia* in this treatment combination evolved muted thermal plasticity compared to *Daphnia* in the “cold‐evolved algae + cold‐selected *Daphnia*” combination. If thermal plasticity confers fitness benefits, then rapid thermal adaptation in resources could lead to maladaptation in consumers.

### 4.5 Effect of predators on *Daphnia* thermal evolution: “top‐down” effects

We predicted that the presence of predators would modify *Daphnia* TRN evolution (Box , Hypothesis 3). We did not observe the predicted increase in *Daphnia r* in response to predators, but *Daphnia* did evolve larger bodies, especially those in the 12°C selection treatment. These results are consistent with a previous study showing the evolution of larger bodies and earlier reproduction in the same predator–prey system (Spitze, 1991). However, another study that used the same study system, but with food limitation, did not observe the evolution of larger *Daphnia* body size in the presence of *Chaoborus* predators (Tseng & O'Connor, 2015).

## 5 CONCLUSIONS

Using laboratory experiments on a tri‐trophic aquatic community, we have demonstrated that species interactions can play an important role in determining how a focal population response to temperature‐mediated selection. The challenge moving forward is to apply these results to natural settings and to assess how frequently species interactions hinder or help focal populations adapt to warming climates. There are multiple cases in nature where interacting species may respond to the same selection pressure but at very different speeds (e.g., insects and their coevolved viruses (Cory & Myers, 2003), small mammals and their prey (hare/lynx: Krebs, Boonstra, Boutin, & Sinclair, 2001), trees and their seed predators (Mezquida & Benkman, 2014), fish and zooplankton (Vanni, 1986)), and thus, we expect the “bottom‐up” results of this study to be widely applicable across natural food webs.

## CONFLICT OF INTEREST

None declared.

## Data Availability

All raw data are available in Dryad (https://doi.org/10.5061/dryad.d6f6q7p) (Tseng, Bernhardt, & Chila, 2019).
